# Supplementary figures and images for: Synaptic and dendritic architecture of different types of hippocampal somatostatin interneurons
Source: PLoS Biol. 2024 Mar 12;22(3):e3002539. doi: 10.1371/journal.pbio.3002539 (PMC10959371; doi:10.1371/journal.pbio.3002539)

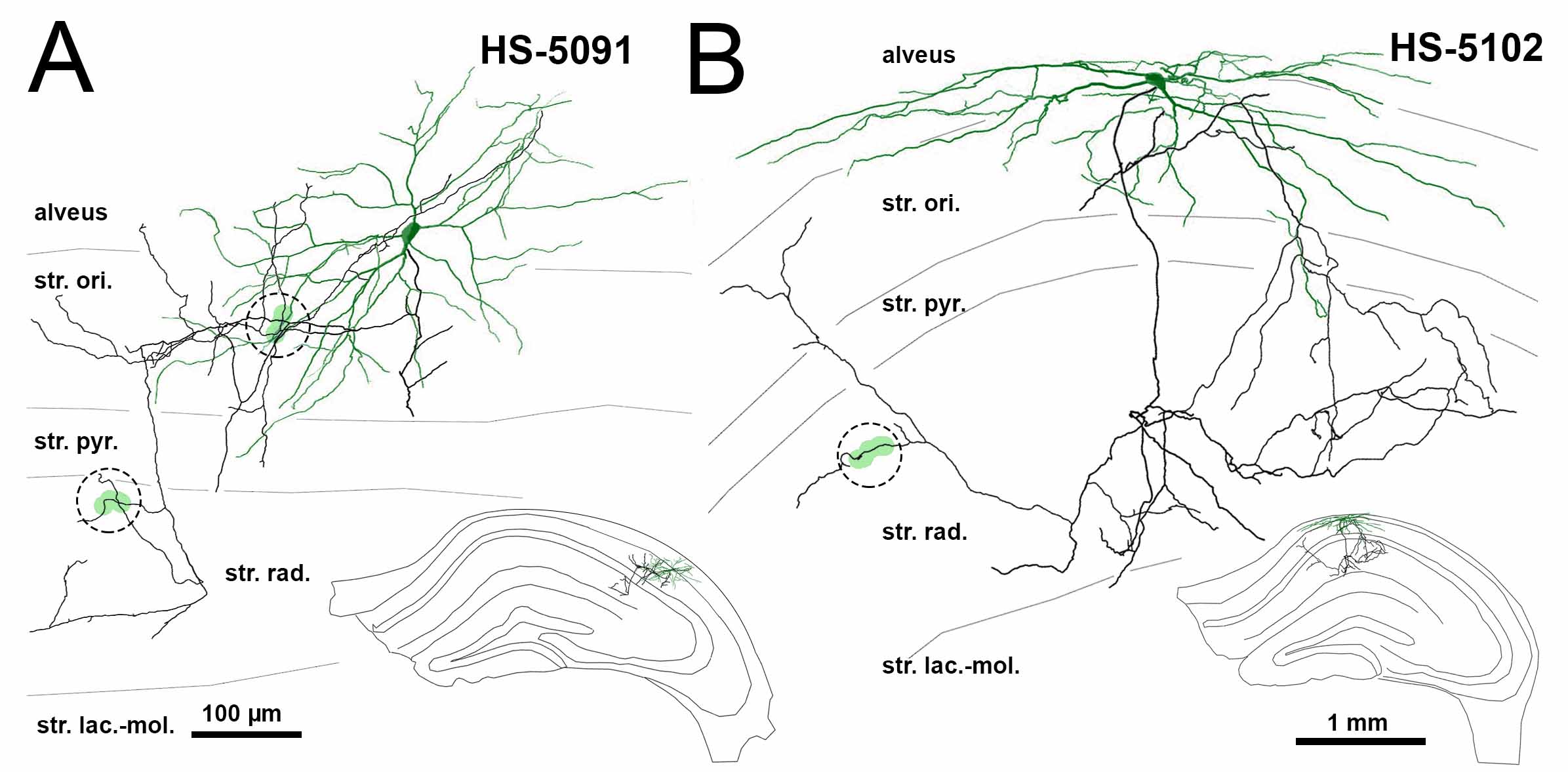

Supplement: S1 Fig — Neurolucida reconstructions of BDA-labeled HS cells. Complete dendritic trees are shown in green, whereas partially reconstructed local axons are shown in black. Inset shows the location of cells in str. oriens of the hippocampal CA1 area. The axonal segments indicated with green highlighting and dashed circles were reconstructed and analyzed in 3D using a scanning electron microscope. (JPG) [file pbio.3002539.s001.jpg]

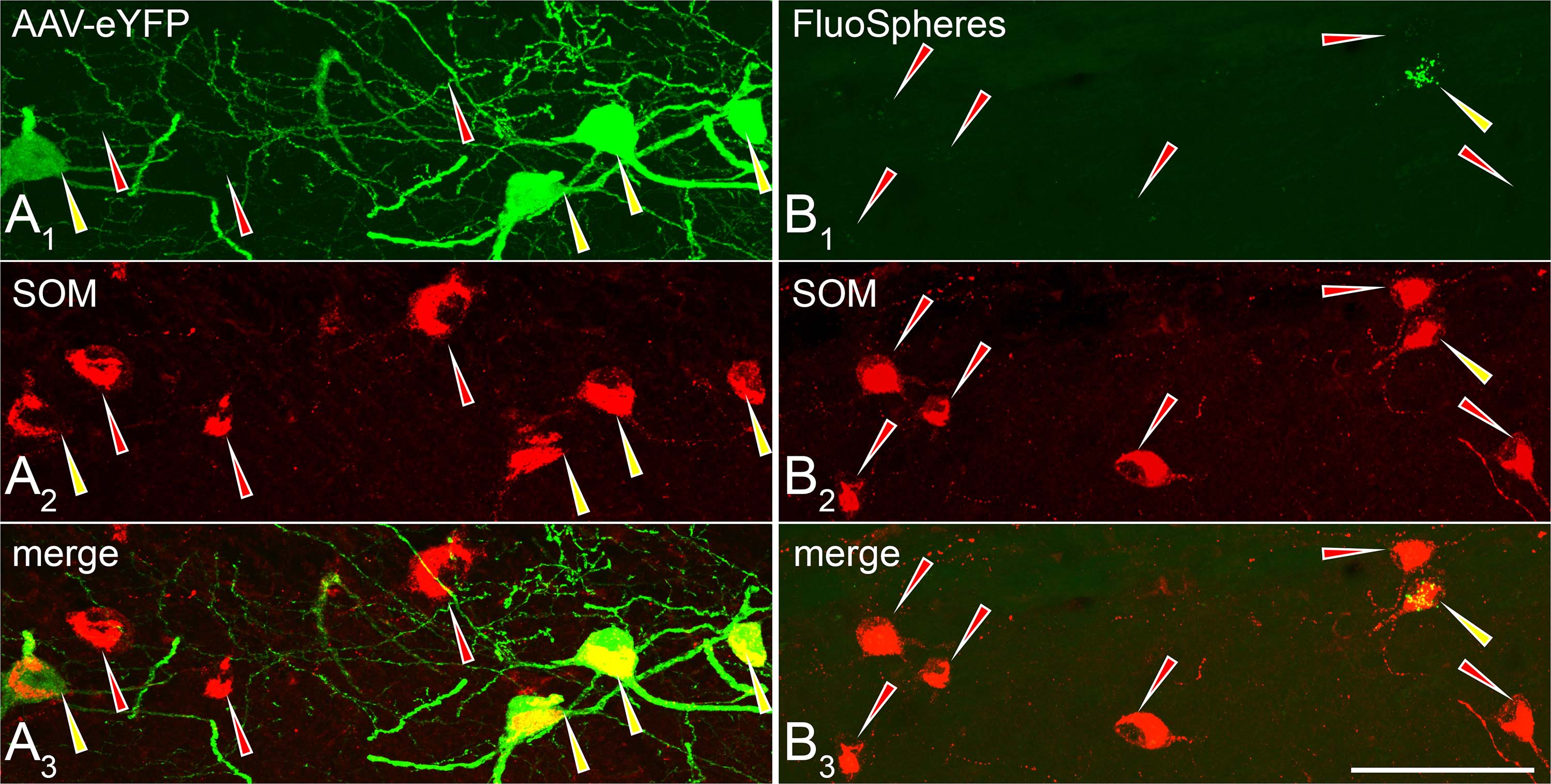

Supplement: S2 Fig — A1-3: Confocal laser-scanning microscope image of a Cre-dependent AAV-eYFP tracer injection site in the CA1 str. oriens of a Chrna2-Cre mouse. The section was double-labeled for somatostatin and eYFP, and 49.1%, 42%, and 37.9% of somatostatin-positive cells were eYFP-positive OLM cells in 3 mice, respectively. B1-3: Confocal laser-scanning microscope image from CA1 str. oriens in a mouse with FluoSpheres-injection into the medial septum. Yellow-green fluorescent microbeads were retrogradely transported into the somata of HS cells, whereas somatostatin was labeled by immunocytochemistry; 9.5%, 8.1%, and 7.5% of somatostatin-positive cells were tracer-containing HS cells in 3 mice, respectively. Yellow arrowheads indicate double-labeled cells, whereas red arrowheads show somatostatin-positive cells that did not contain tracer. Scale bar: 50 μm. (JPG) [file pbio.3002539.s002.jpg]

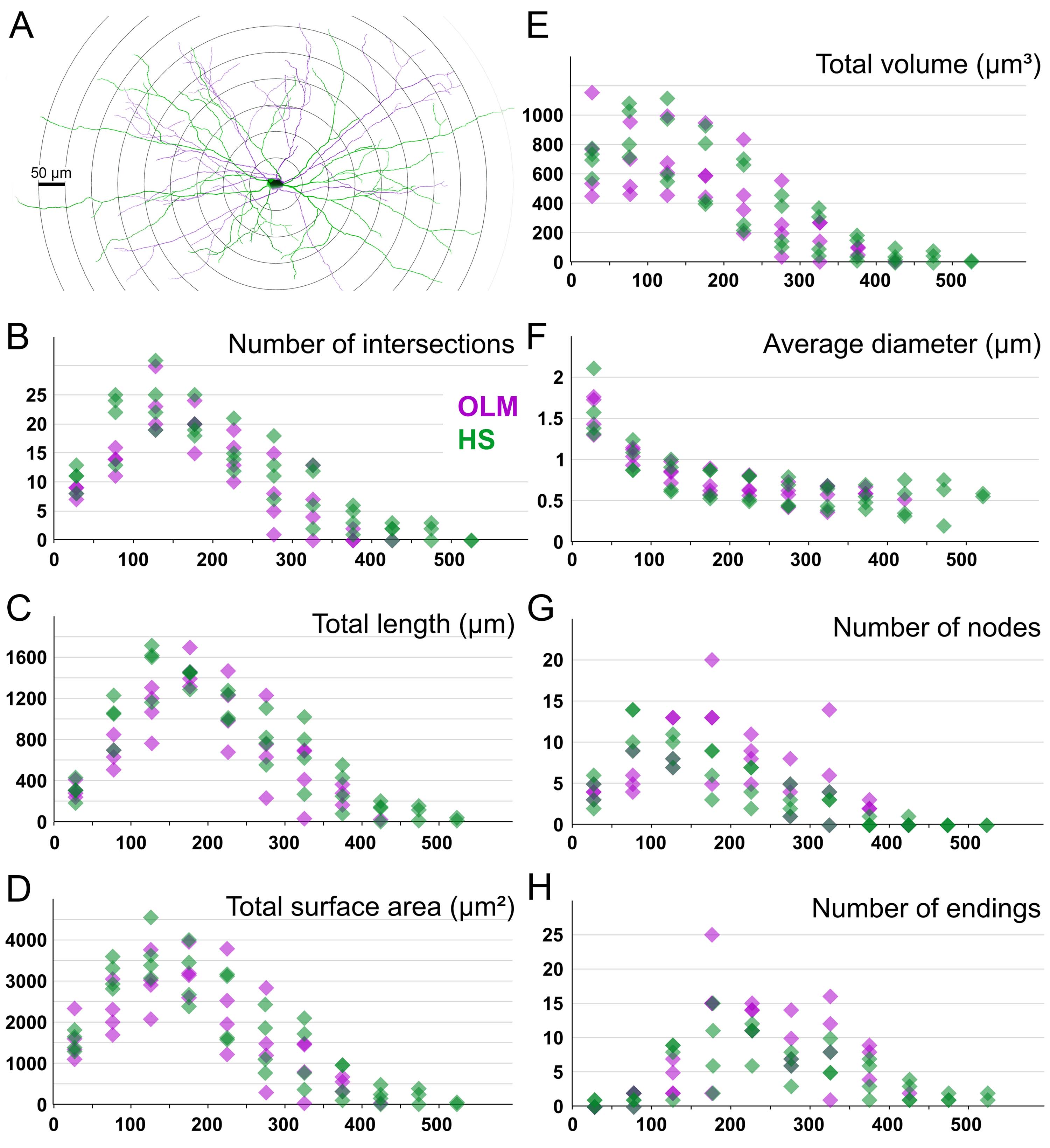

Supplement: S3 Fig — (A) Dendritic trees of an OLM (magenta) and an HS cell (green) with overlaid concentric spheres. The radius increment of each sphere is 50 μm. (B–H) Morphological parameters of OLM (magenta) and HS cells (green) measured 3 dimensions in concentric spherical shells 50–550 μm from soma. Data points represent single cells. (B) Number of dendrites intersecting a sphere. (C) Total dendritic length in shells. (D) Total surface area of dendrites in shells. (E) Total volume of dendrites in shells. (F) Average diameter of dendrites in shells. (G) Number of branching points (nodes) in shells. (H) Number of dendrites ending in a shell. The analysis did not reveal differences, except that HS cells had branches in shells further than 500 μm, while the dendrites of the OLM cells have not reached that far from their somata. See raw data (exported directly from Neurolucida Explorer) in S20 Data. (JPG) [file pbio.3002539.s003.jpg]

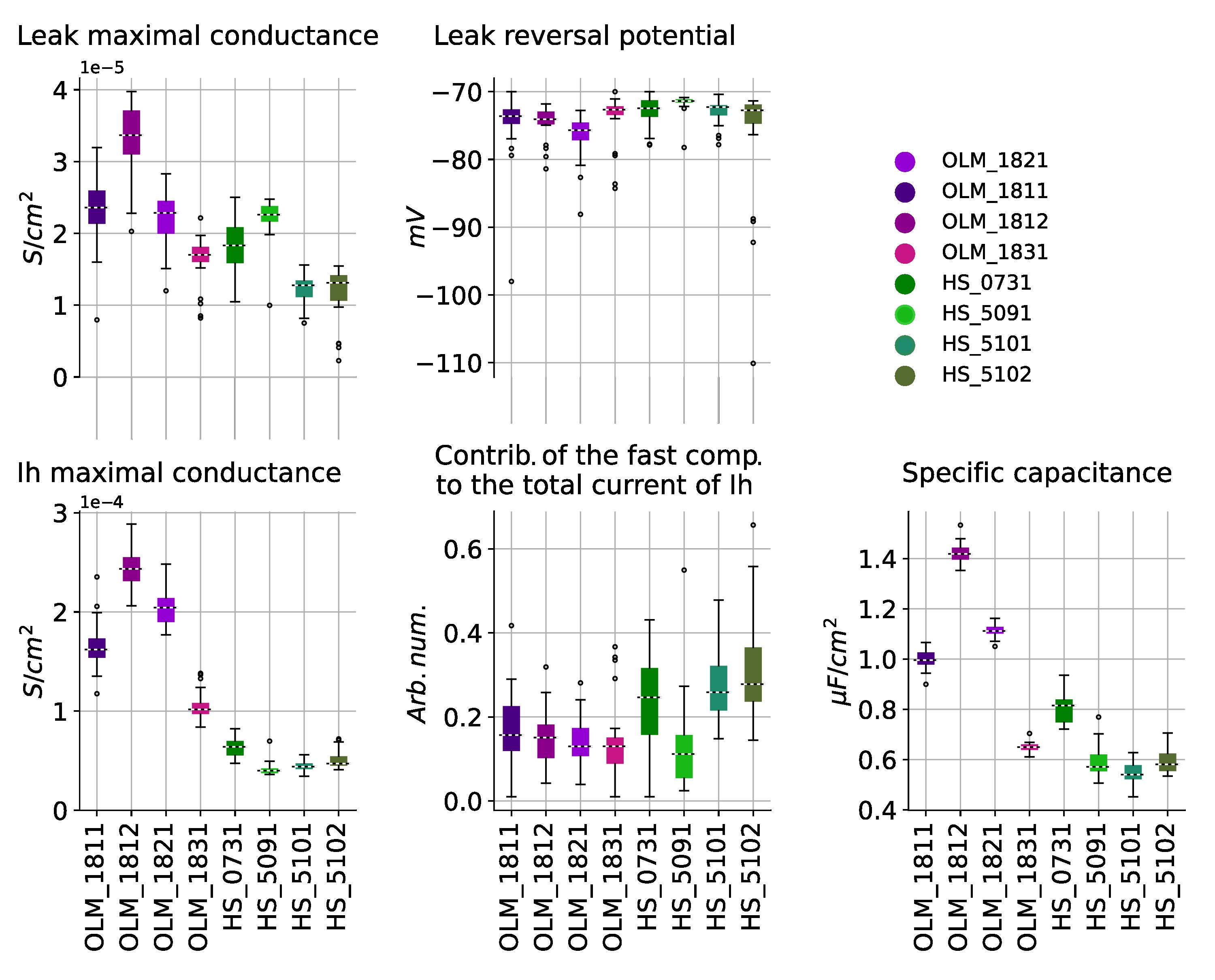

Supplement: S4 Fig — The median and interquartile values (q1: 25%, q2: 75%) of the best-fitting parameters of the 20 optimization runs for each cell morphology are indicated by the boxes. Whiskers extend to the smallest and largest value within the kernel of q1-1.5* interquartile range and q2+1.5* interquartile range. Those values that are not in that kernel are defined as outliers and represented as circles. See raw data in S24 Data. (JPG) [file pbio.3002539.s004.jpg]

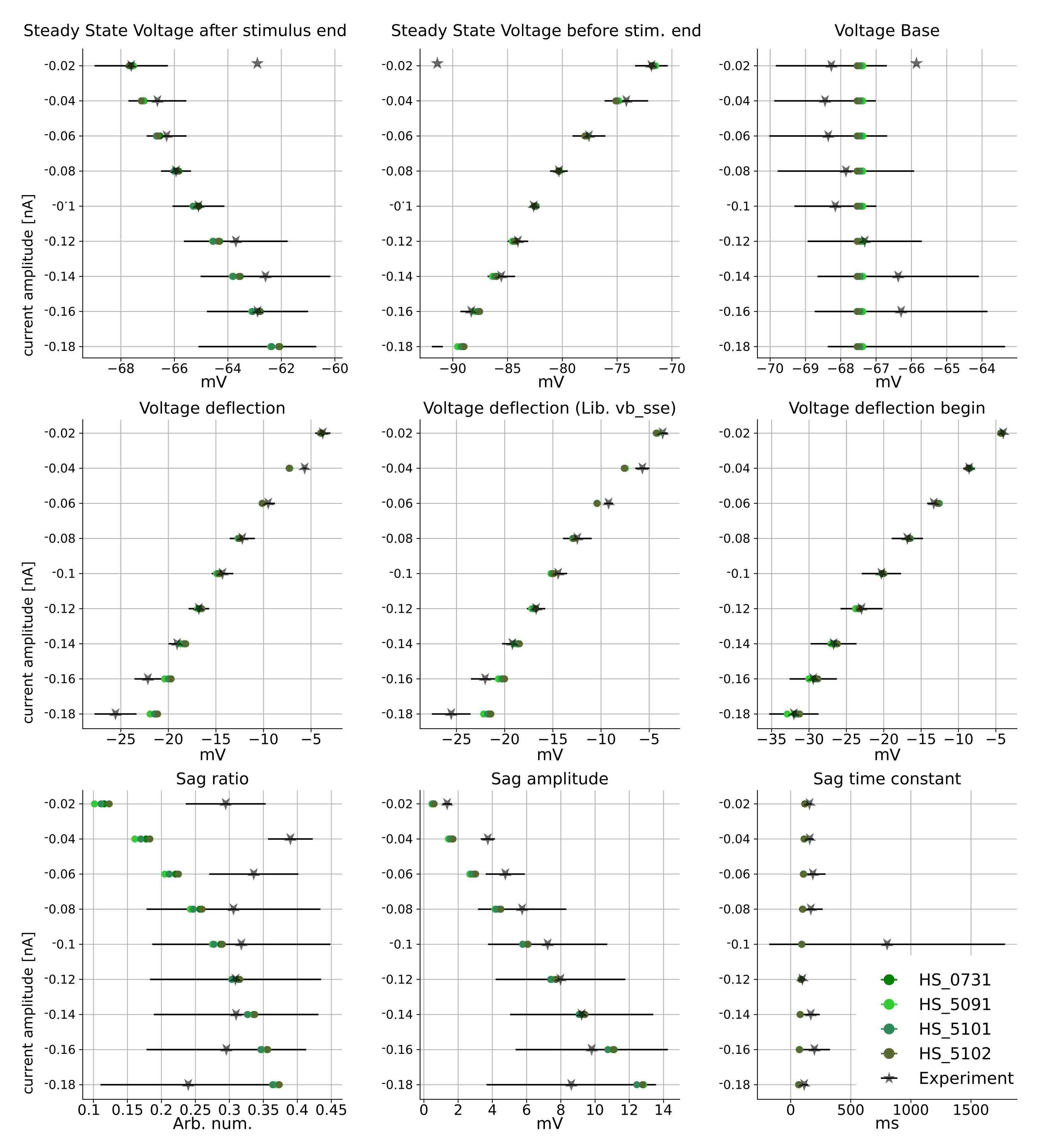

Supplement: S5 Fig — The mean of the experimental data is marked with black X with its corresponding standard deviation. Each cell is represented by its associated color. Only the features corresponding to hyperpolarizing current injections were used in this validation. In the Electrophys Feature Extraction Library (eFEL), the exact feature names are the following: Steady state voltage after stimulus end: steady_state_voltage; Steady state voltage before stimulus end: steady_state_voltage_stimend; Voltage base: voltage_base; Voltage deflection: voltage_deflection; Voltage deflection (Lib.: vs_sse): voltage_deflection_vbsse; Voltage deflection begin (at the beginning of the stimulus): voltage_deflection_begin; Sag ratio: sag_ratio1; Sag amplitude: sag_amplitude; Sag time constant: sag_time_constant. The data for this figure is available in the folder Codes_and_scripts\hippounit\somatic_feature_results\Simplified_models\Chosen_HS_somafeatures_optimized at https://zenodo.org/records/10580838. (JPG) [file pbio.3002539.s005.jpg]

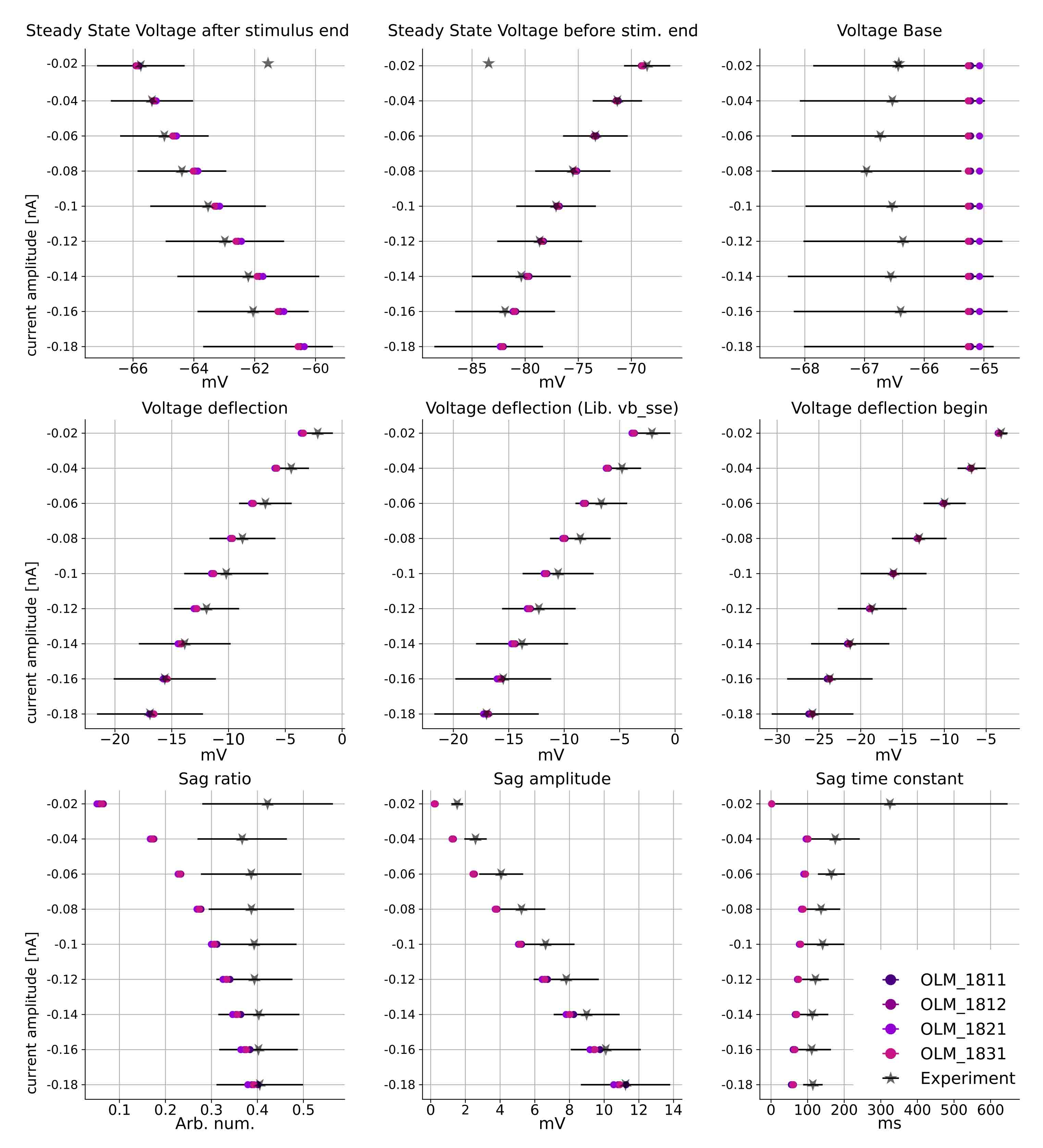

Supplement: S6 Fig — The mean of the experimental data is marked with black X with its corresponding standard deviation. Each cell is represented by their associated color. Only the features corresponding to hyperpolarizing current injections were used in this validation. In the Electrophys Feature Extraction Library (eFEL), the exact feature names are the following: Steady state voltage after stimulus end: steady_state_voltage; Steady state voltage before stimulus end: steady_state_voltage_stimend; Voltage base: voltage_base; Voltage deflection: voltage_deflection; Voltage deflection (Lib.: vs_sse): voltage_deflection_vbsse; Voltage deflection begin (at the beginning of the stimulus): voltage_deflection_begin; Sag ratio: sag_ratio1; Sag amplitude: sag_amplitude; Sag time constant: sag_time_constant. The data for this figure is available in the folder Codes_and_scripts\hippounit\somatic_feature_results\Simplified_models\Chosen_OLM_somafeatures_optimized at https://zenodo.org/records/10580838. (JPG) [file pbio.3002539.s006.jpg]

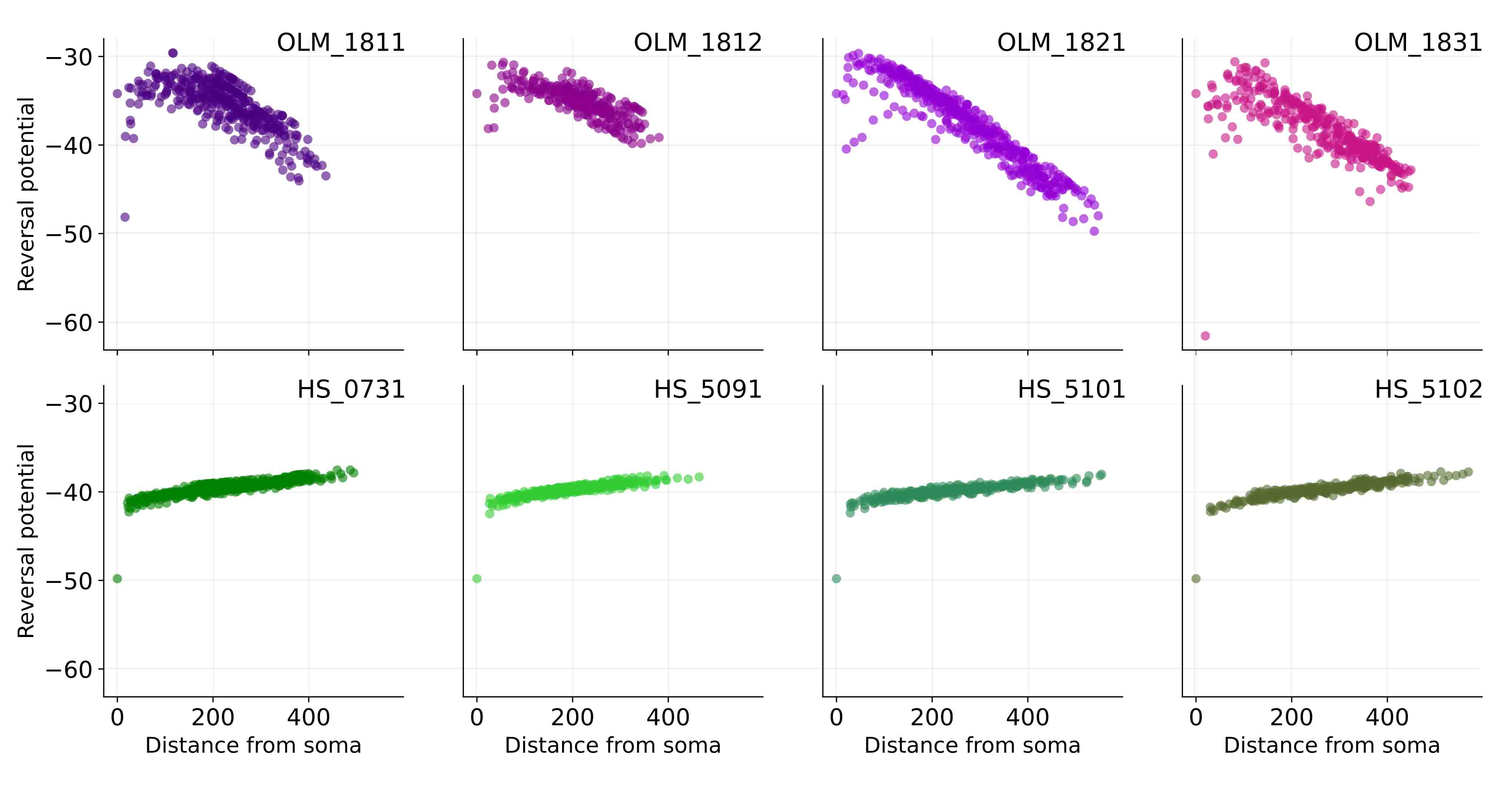

Supplement: S7 Fig — The local reversal potential of dendritic segments as a function of their distance from the soma, calculated from the estimated average excitatory, inhibitory, and leak currents in the high-conductance state of each passive model neuron. (JPG) [file pbio.3002539.s007.jpg]

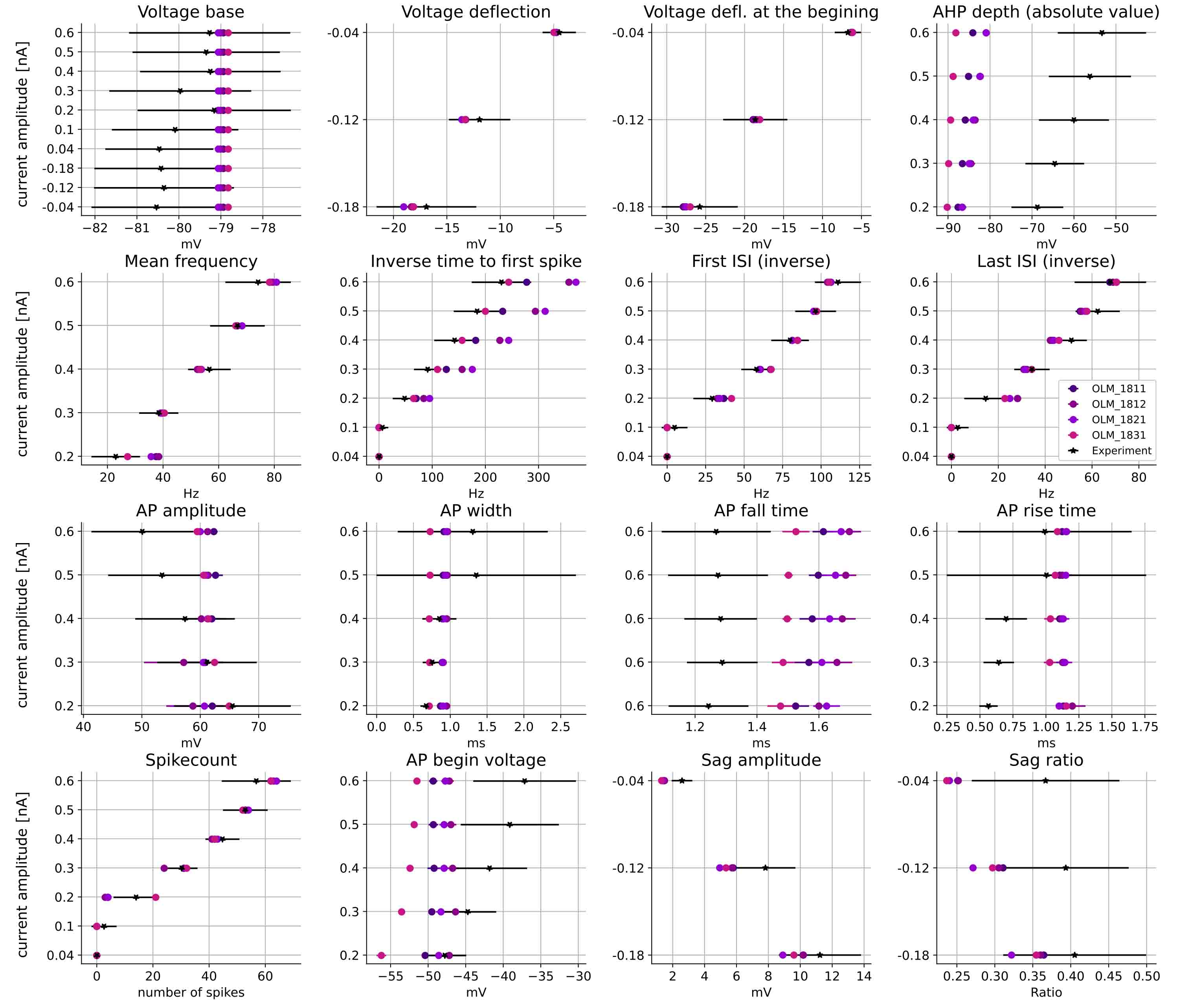

Supplement: S8 Fig — The mean of the experimental data is marked with black X with its corresponding standard deviation. Each cell is represented by its associated color. Sixteen features were selected for this figure to represent both spiking and non-spiking behavior of the cell without redundancy from the HippoUnit’s Somatic Features Test. The data for this figure is available in the folder Codes_and_scripts\hippounit\somatic_feature_results\Detailed_models\Validation_chosen_ones_olm at https://zenodo.org/records/10580838. (JPG) [file pbio.3002539.s008.jpg]

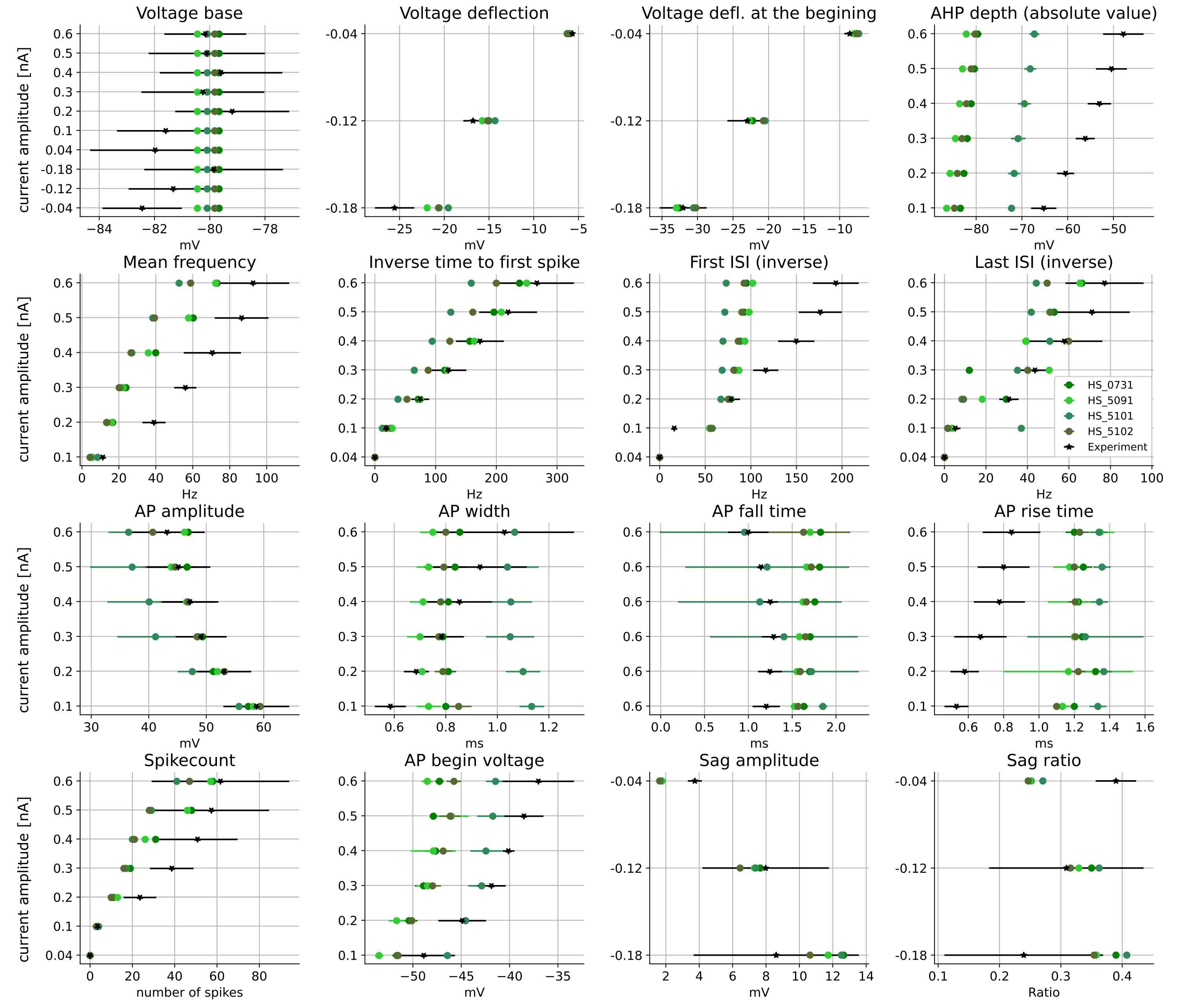

Supplement: S9 Fig — The mean of the experimental data is marked with black X with its corresponding standard deviation. Each cell is represented by its associated color. Sixteen features were selected for this figure to represent both spiking and non-spiking behavior of the cell without redundancy from the HippoUnit’s Somatic Features Test. The data for this figure is available in the folder Codes_and_scripts\hippounit\somatic_feature_results\Detailed_models\Validation_chosen_ones_hs at https://zenodo.org/records/10580838. (JPG) [file pbio.3002539.s009.jpg]

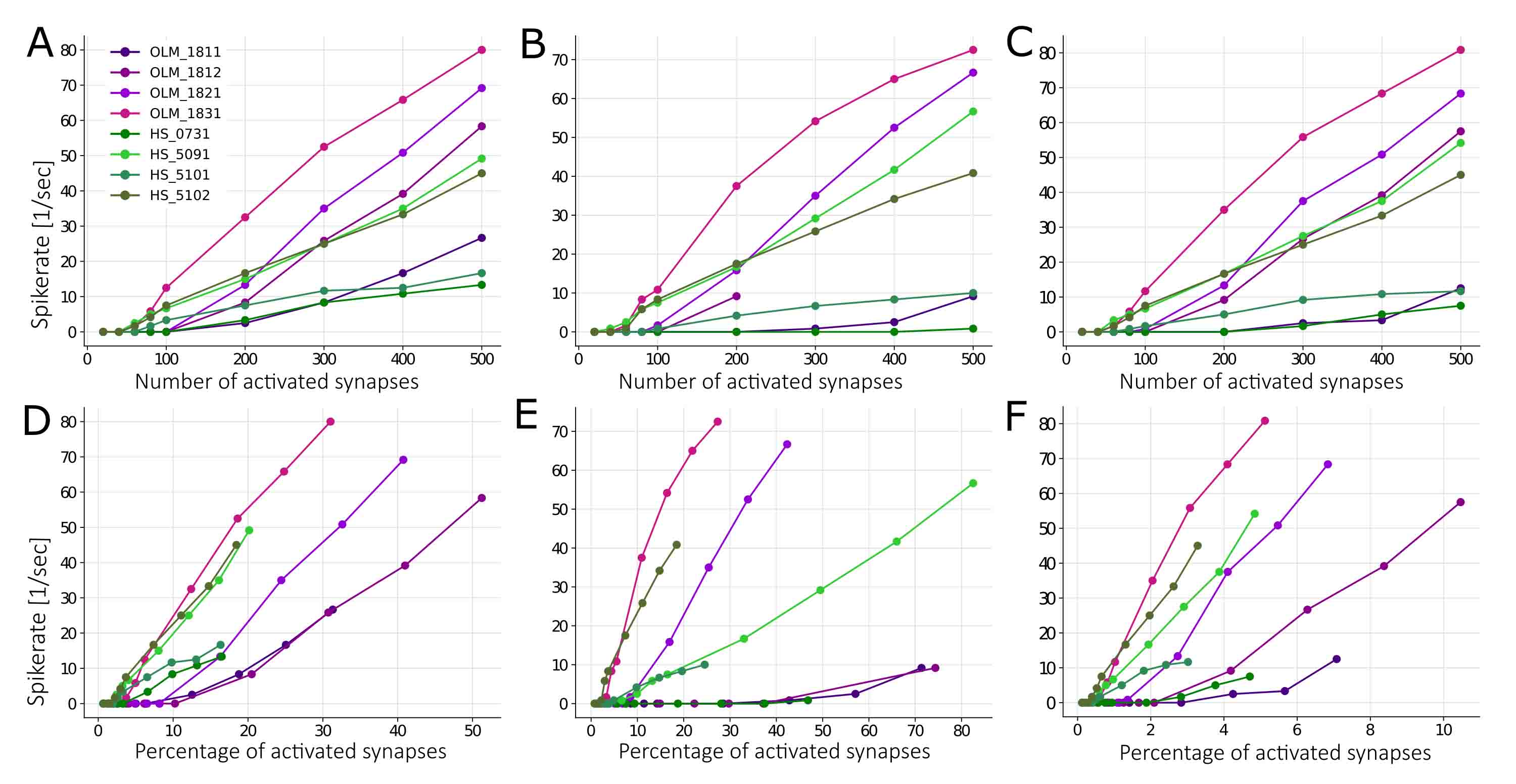

Supplement: S10 Fig — (A–C) The spiking rates of model neurons as a function of the number of activated synaptic inputs. (D–F) The same data shown as a function of the percentage of activated synapses in the given cell and distance range. Synapses were selected randomly from different distance ranges from the soma: (A, D) 0–100 μm; (B, E) 300–400 μm; (C, F) 0–400 μm. (JPG) [file pbio.3002539.s010.jpg]

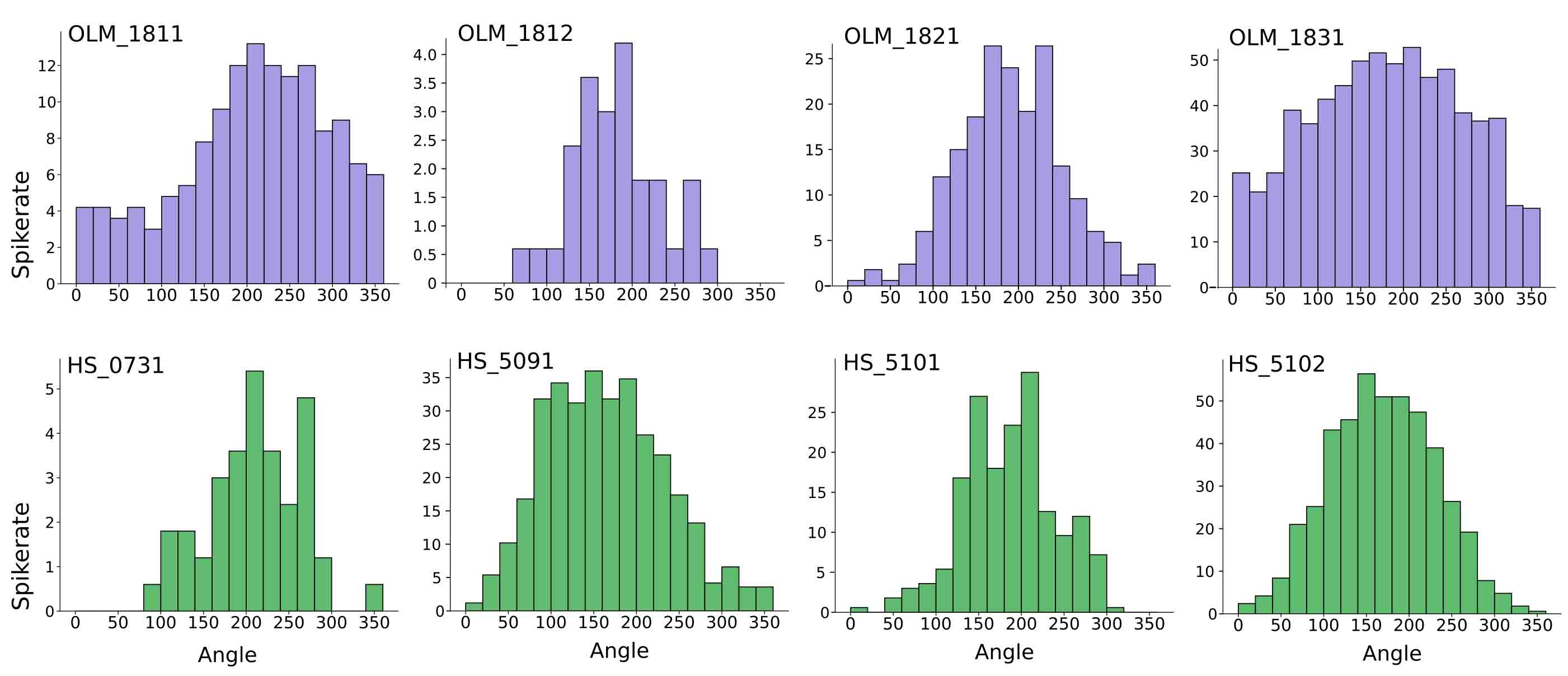

Supplement: S11 Fig — Spike rates of active model cells as a function of the phase (in degrees) of the 7 Hz theta oscillation that modulated the rate of synaptic inputs; 5% of the total number of excitatory synapses were randomly distributed in the whole dendritic arbor in all cells except OLM_1811, where the percent of activated synapses was increased to 15% to ensure sufficient activity. See raw data in S25 Data. (JPG) [file pbio.3002539.s011.jpg]

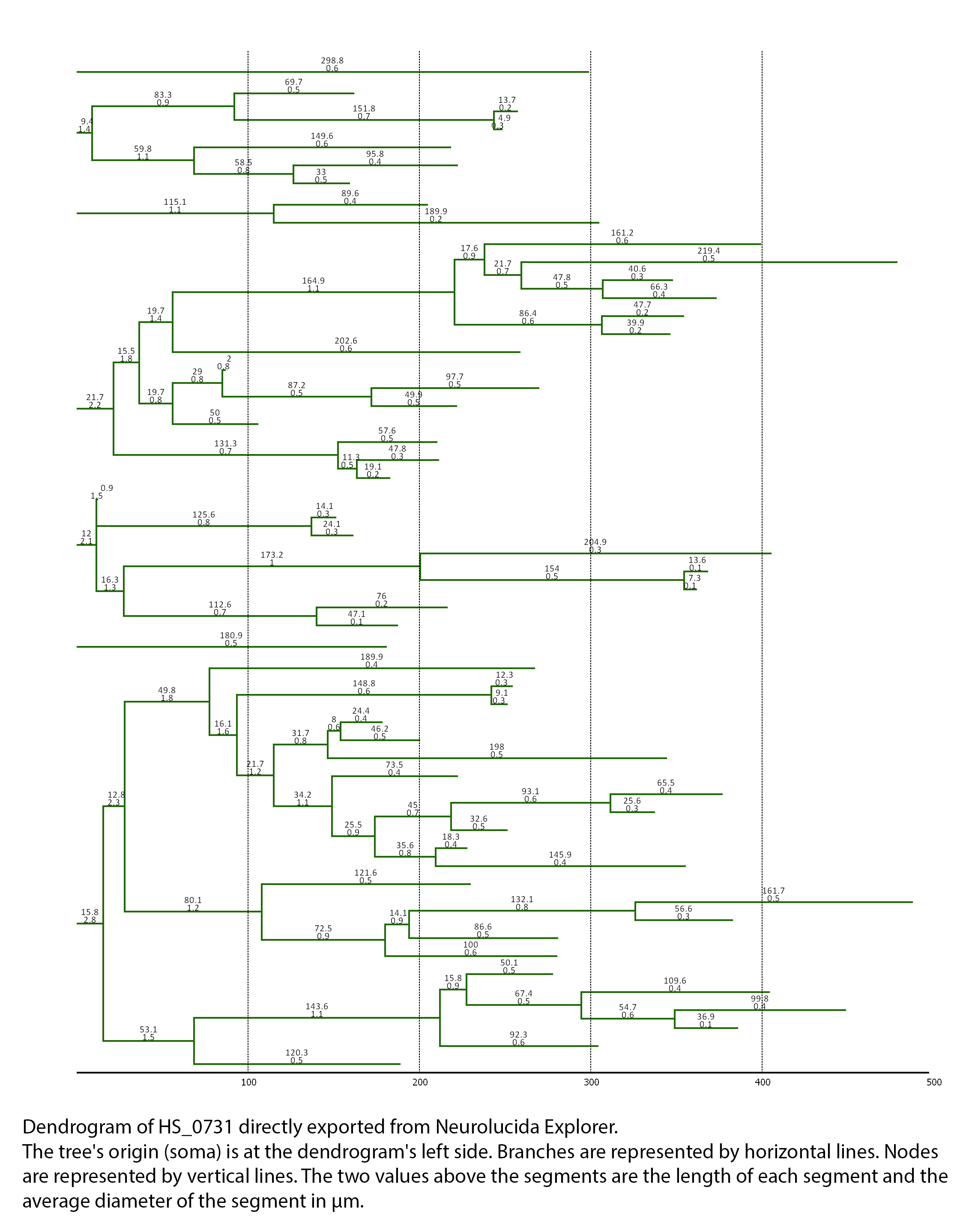

Supplement: S22 Data — (ZIP) [file pbio.3002539.s033.zip › Dendrogram_HS_0731.jpg]

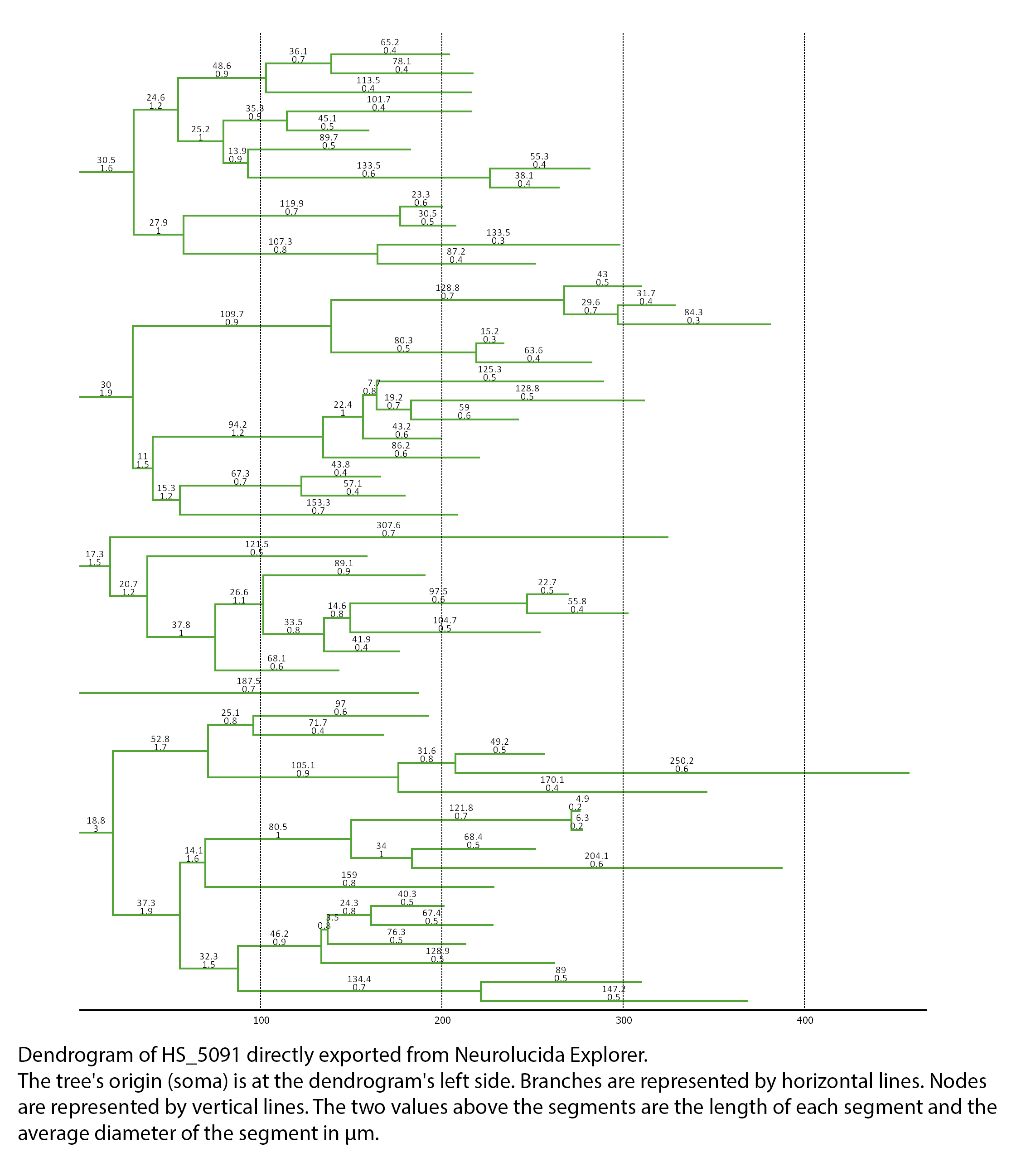

Supplement: S22 Data — (ZIP) [file pbio.3002539.s033.zip › Dendrogram_HS_5091.jpg]

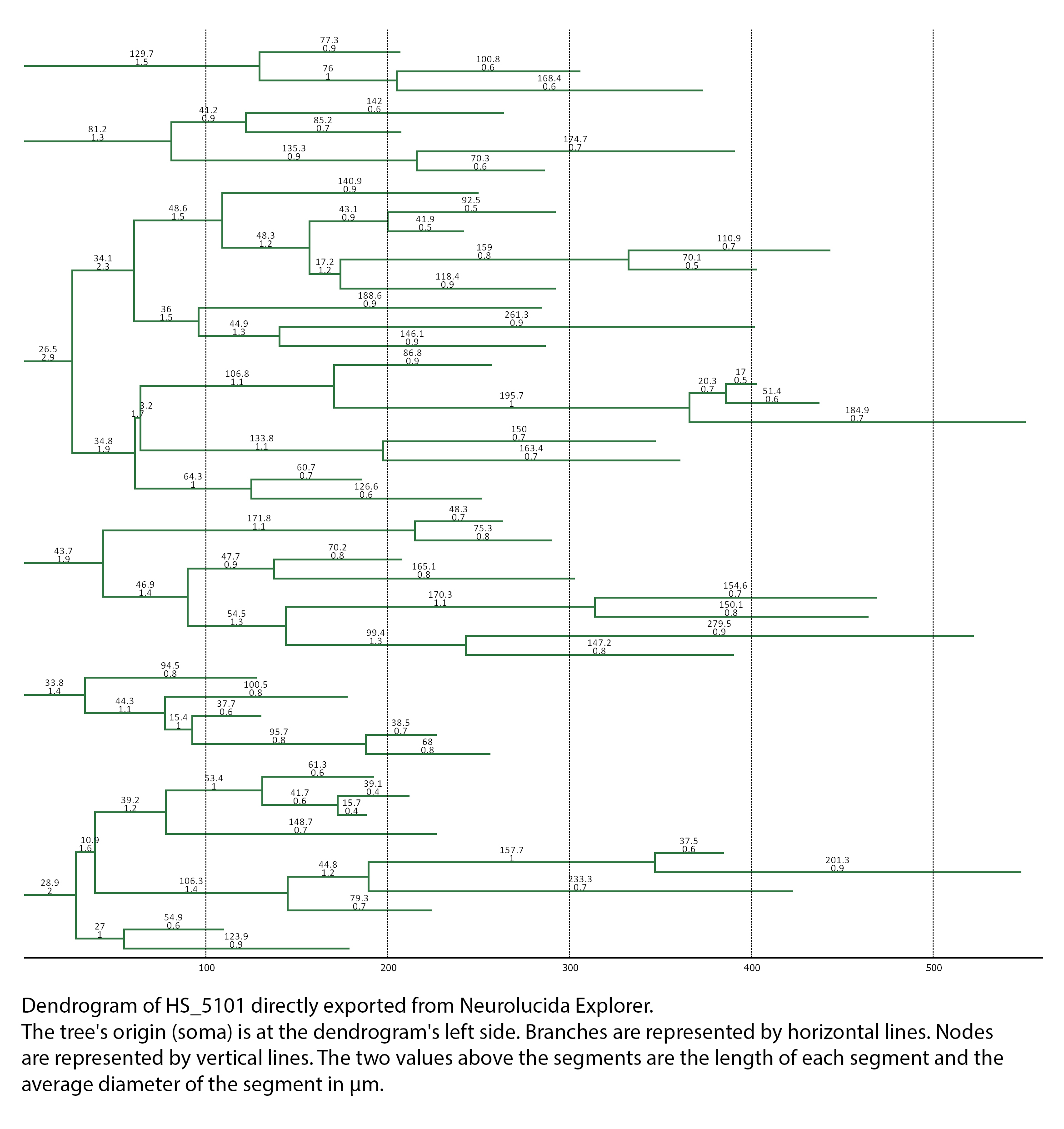

Supplement: S22 Data — (ZIP) [file pbio.3002539.s033.zip › Dendrogram_HS_5101.jpg]

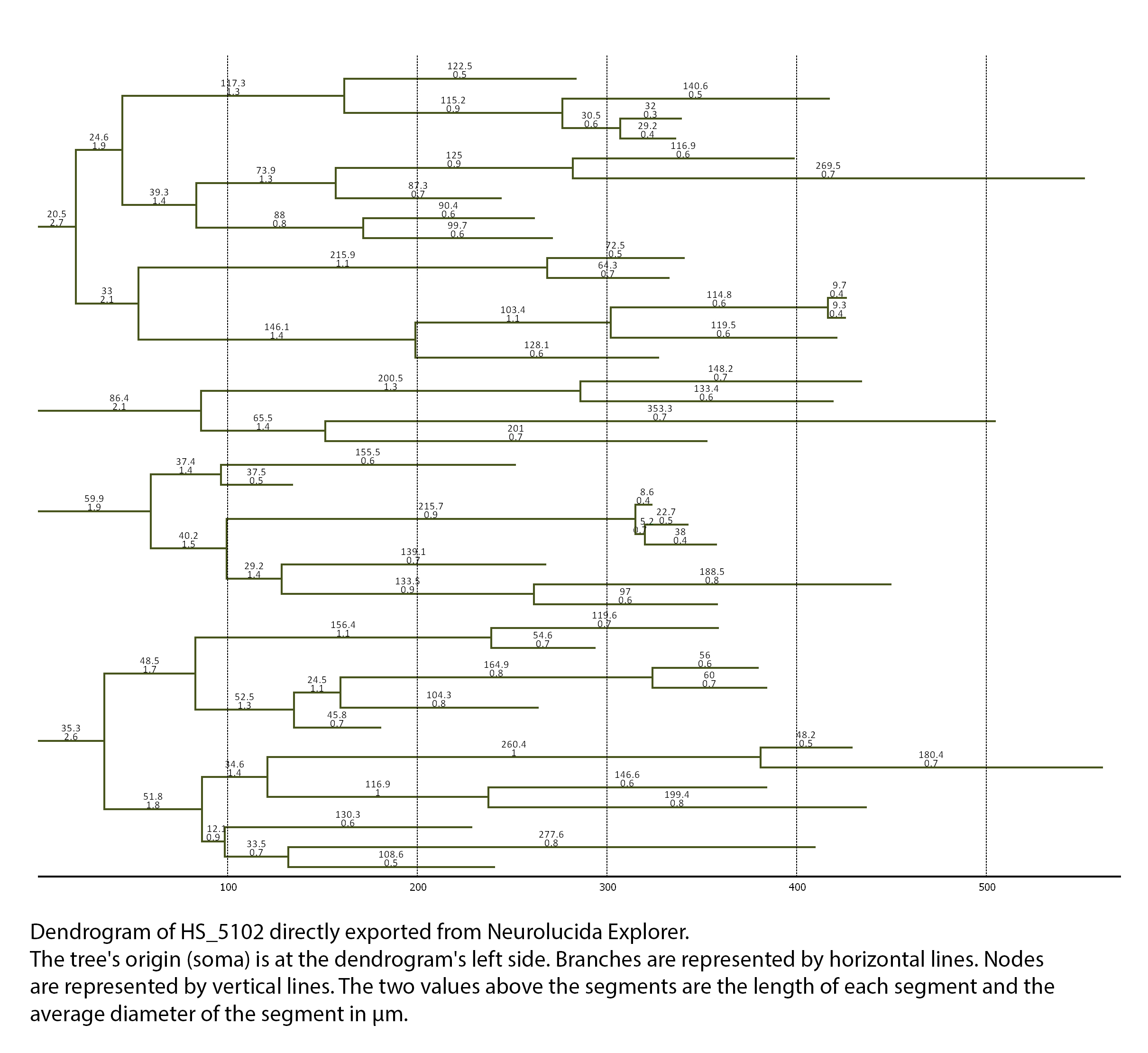

Supplement: S22 Data — (ZIP) [file pbio.3002539.s033.zip › Dendrogram_HS_5102.jpg]

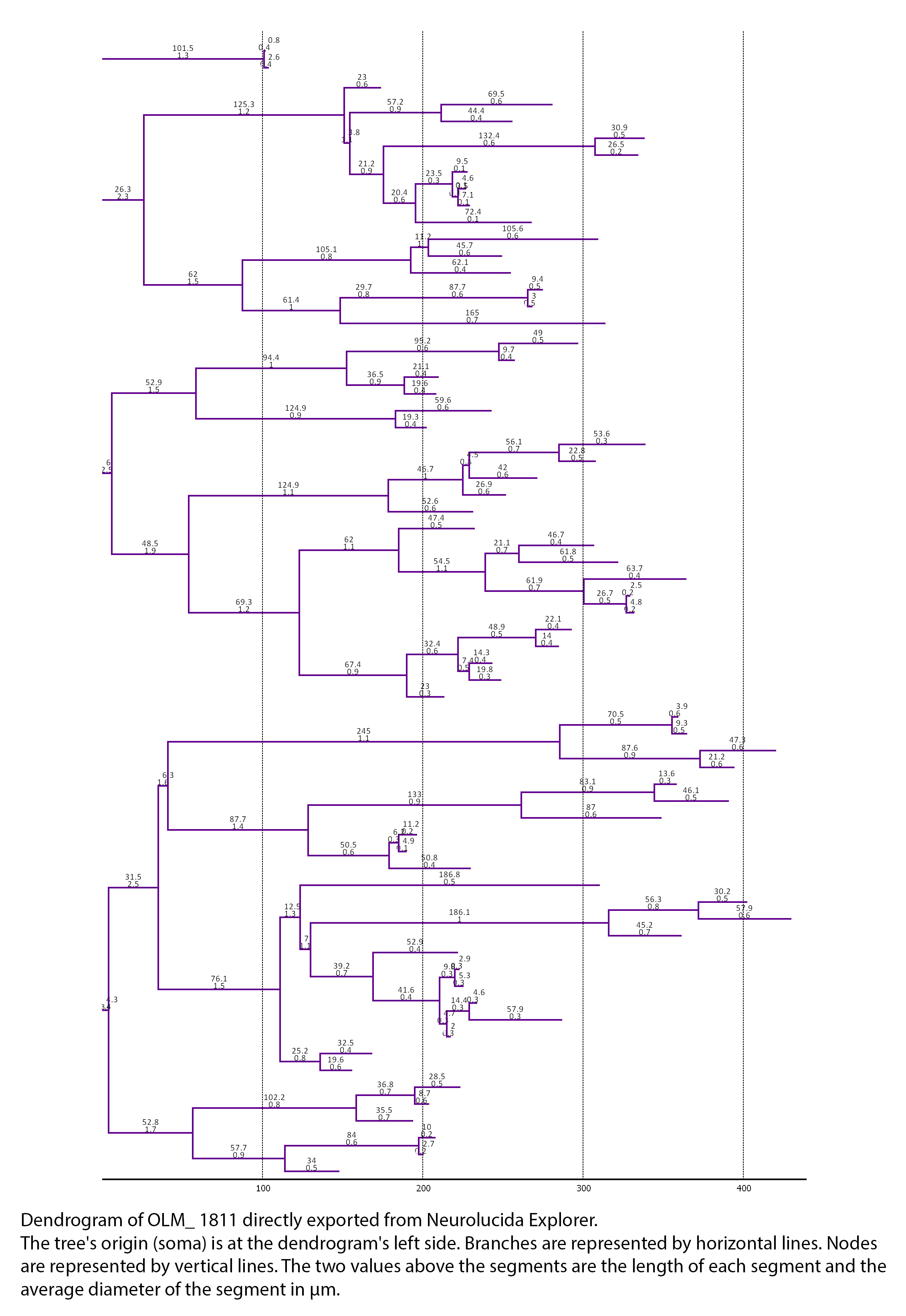

Supplement: S22 Data — (ZIP) [file pbio.3002539.s033.zip › Dendrogram_OLM_1811.jpg]

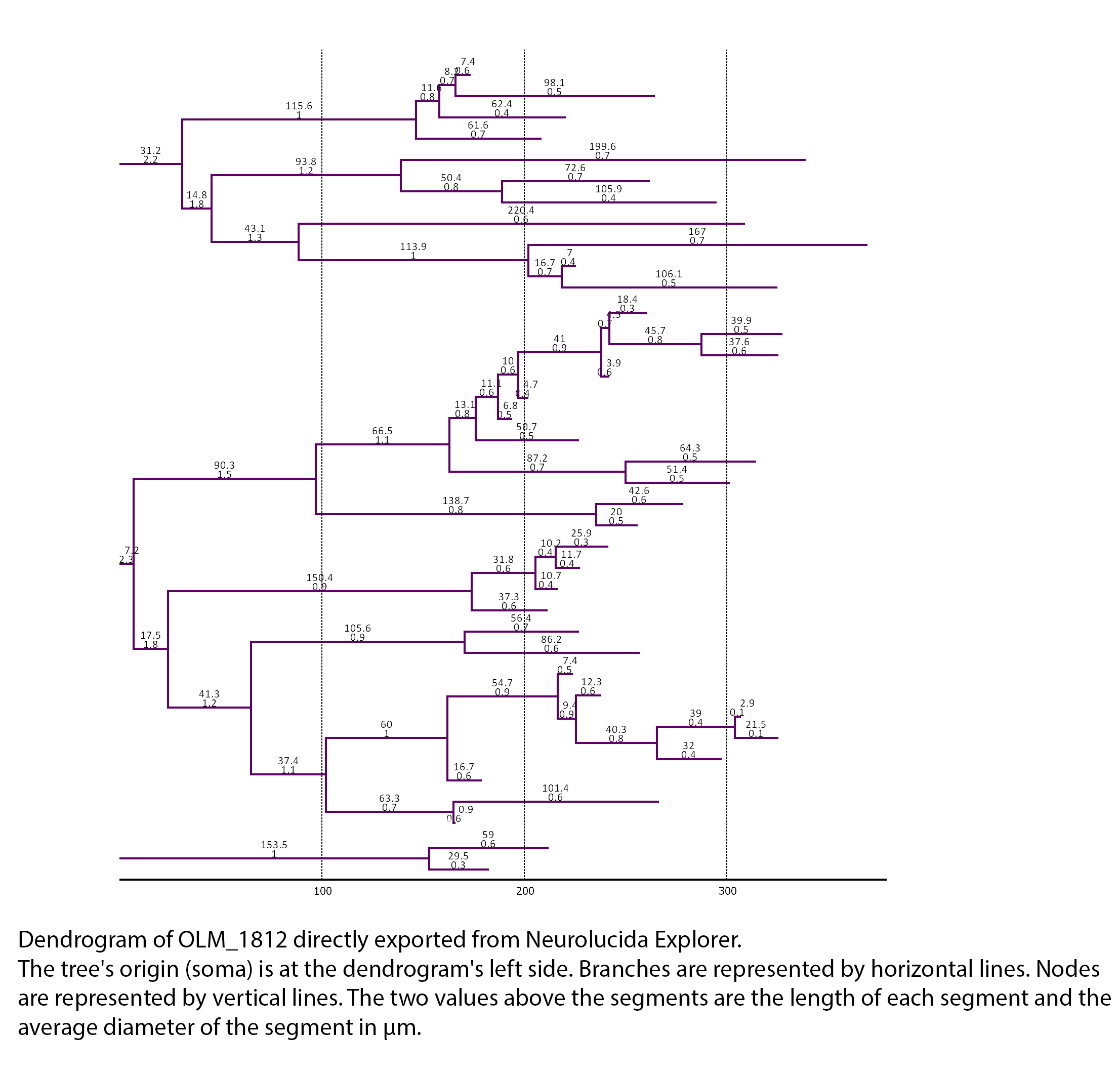

Supplement: S22 Data — (ZIP) [file pbio.3002539.s033.zip › Dendrogram_OLM_1812.jpg]

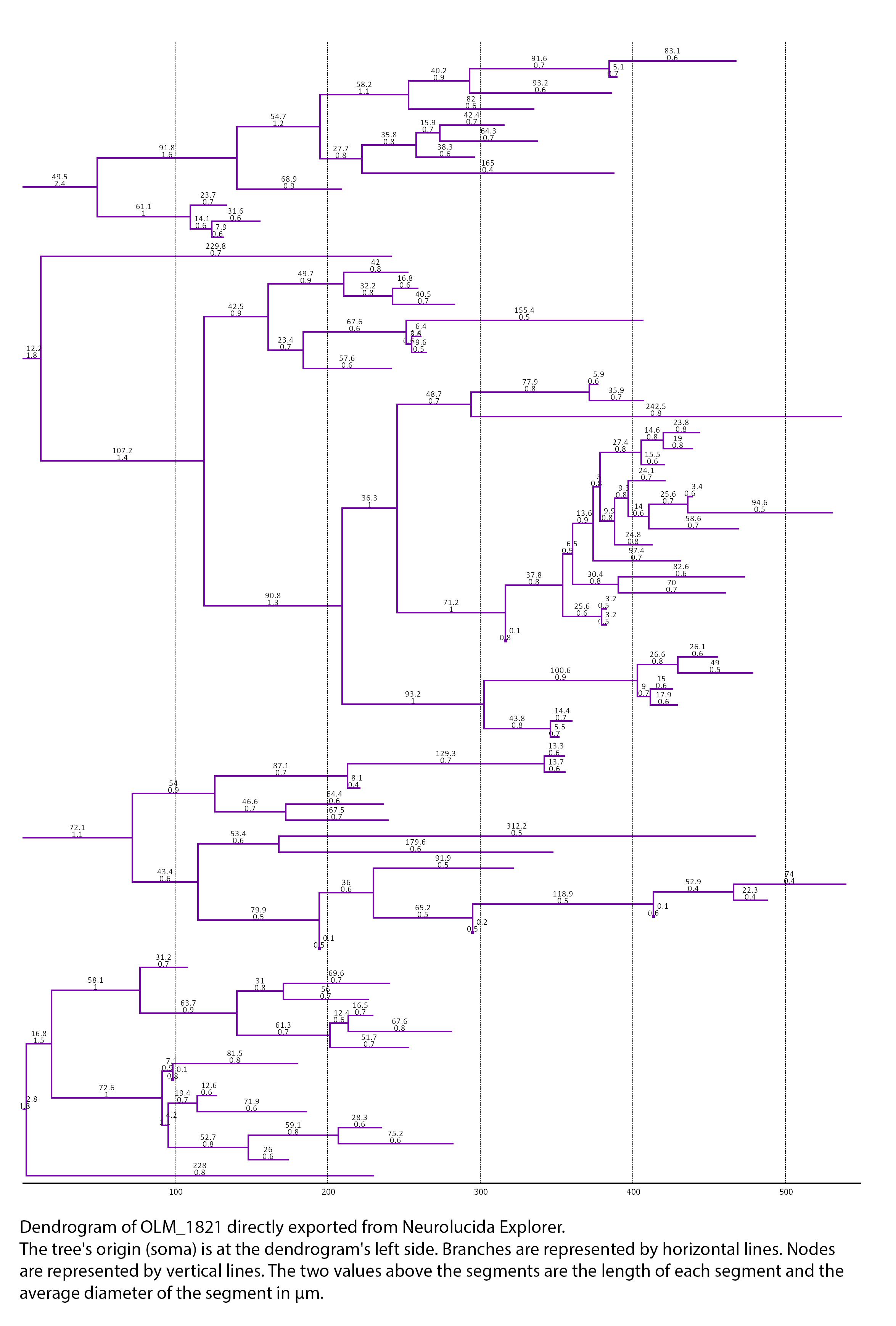

Supplement: S22 Data — (ZIP) [file pbio.3002539.s033.zip › Dendrogram_OLM_1821.jpg]

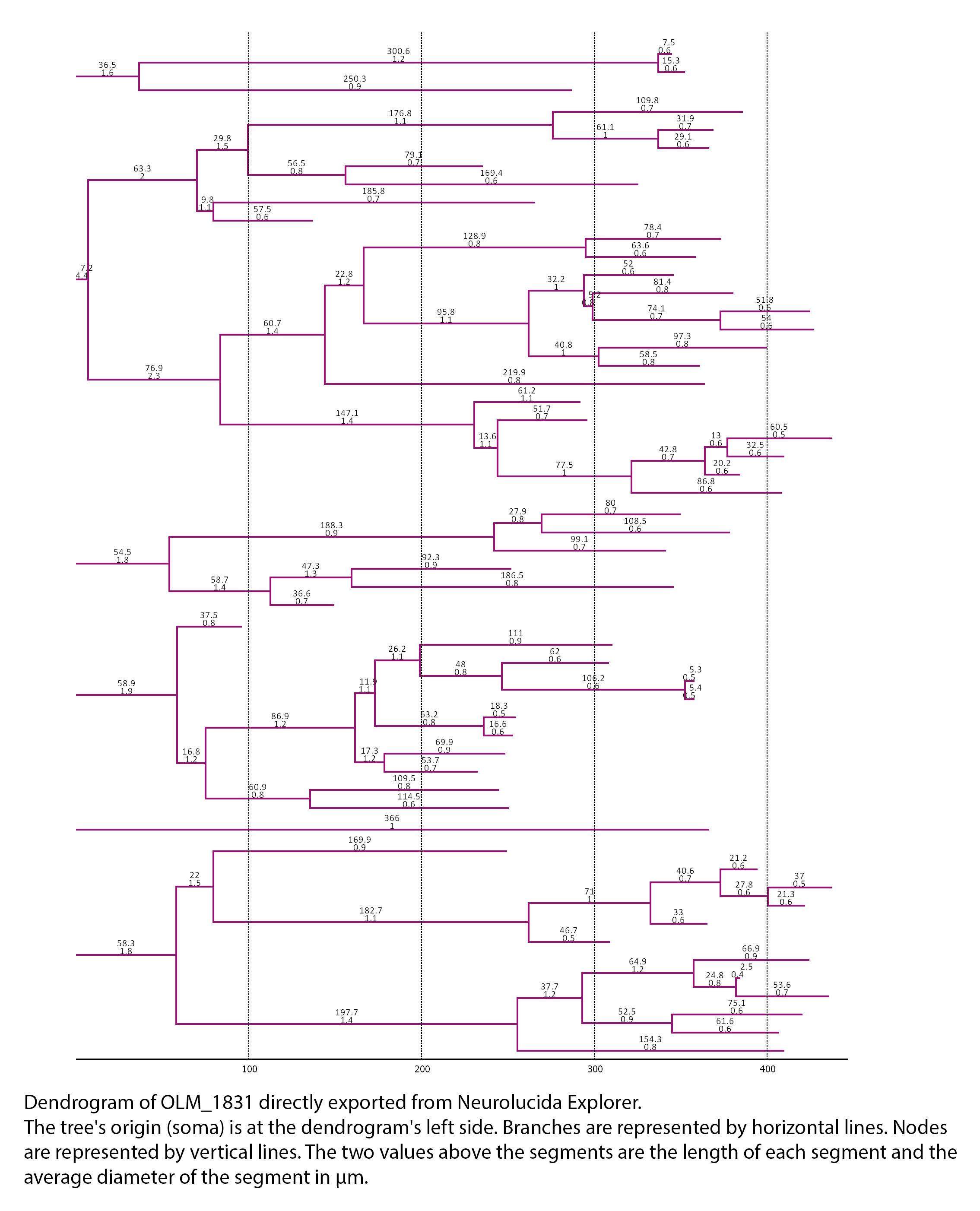

Supplement: S22 Data — (ZIP) [file pbio.3002539.s033.zip › Dendrogram_OLM_1831.jpg]
