# Supplementary material for: Synaptic and dendritic architecture of different types of hippocampal somatostatin interneurons
Source: PLoS Biol. 2024 Mar 12;22(3):e3002539. doi: 10.1371/journal.pbio.3002539 (PMC10959371; doi:10.1371/journal.pbio.3002539)
Supplement: Attachment — Submitted filename: Takacs_etal_Response to Reviewers.pdf [file pbio.3002539.s037.pdf]

**Response to comments by the Reviewers**

Methods and Resources Article for PLOS Biology ID: PBIOLOGY-D-23-01714R1
Title: Synaptic and dendritic architecture of two types of hippocampal somatostatin interneurons

*We appreciate the Reviewers' constructive feedback and their positive comments on our manuscript. We have now carried out further analysis and modeling, created several new datasets and figures, and updated the manuscript accordingly. The most significant addition is the creation and simulation of fully active models of OLM and HS cells. Using these new models, we examined the predicted action potential output of the two cell types in response to synaptic inputs, varying the number and location of the inputs. We also looked at how the spike output of the models is modulated by scattered synaptic input whose rate varies at the theta frequency. As per the Reviewer's suggestions, we have also conducted additional anatomical measurements.*

*Below we present the entire set of reviewer comments in full (in black text), together with our point-by-point responses (in blue text). In the manuscript, we have also indicated in blue text the changes that relate to the Reviewers' suggestions.*

**Reviewer: 1**

The work by Takács and colleagues is detailed quantitative study of the morphological and physiological characteristics, distribution and size of putative glutamatergic excitatory and GABAergic inhibitory synaptic inputs along the entire somato-dendritic domain of Hl and OLM cells in CA1. The main findings are: (1) dendrites were highly variable in thickness in both Hl than OLM cells; (2) the density and number of glutamatergic and GABAergic inputs was higher in HL than OLM cells; (3) synaptic inputs were larger at proximal than distal dendrites of HL cells but not OLM cells; in OLM neurons glutamatergic inputs increased in size with distance from the soma; (4) the size of output synapses of OLM cells was almost twice as large as those of HL cells. Finally, HL cells tended to discharge at mildly higher frequency in vitro during current injections, particularly at the beginning of the injection of a given amplitude. On the basis of anatomical reconstructions and estimates on single synaptic as well as total compartment-specific synaptic and leak conductances they further developed single cell computational models to define how the distribution of inputs may shape EPSPs and IPSPs measured at the soma in tow states: the silent and a high conductive state. As expected, EPSPs were larger in size in HL cells at silent states than OLM cells and during high conductance states, PSPs were smaller in Hl than OLM cells due to the higher total membrane conductance and stronger filtering of the signals during their propagation from the induction side to the soma.

The manuscript is very well written and illustrated. The set of data provided in this study is very important for our understanding how neurons of similar / comparable morphology may differentially influence signal propagation. The study shows that details of input size and distribution matter.

*We are grateful to Reviewer 1 for their constructive comments and hope that the additional computational work included in this revision further demonstrates the potential of this large dataset.*

However, my enthusiasm is diminished by the lacking experimental evidences on single input conductances, which could have been measured by e.g. extracellular minimal stimulation along the somato-dendritic axis (dendritic recordings would have been ideal, however, difficult to perform). Moreover, it remains unclear how the different input properties and their distribution may contribute to spatial and temporal signal integration and the control of action potential generation in the two cell types. In other words, what is the functional difference of both cell types, how do the different input distributions and sizes manifest in potential physiological differences in signal integration?

We agree with the Reviewer that these are important questions, and we answer them in detail below.

Main concern:

1. What is the real single input conductance in both cell types? One option would be to stimulate with minimal intensity synaptic inputs (glutamatergic / GABAergic) at distinct distances between soma and dendrite and measure IPSC / EPSC sizes. The idea behind the proposal is to get a bit more realistic estimates on the conductances used in the single cell models.

We agree with the Reviewer that knowing the correct values of synaptic conductances would allow more realistic modeling of how synaptic inputs affect the output of these neurons. As far as we know, the precise values of synaptic conductances that characterize the various input pathways targeting these cells have not been measured. Precise measurement of synaptic conductances would require dendritic patch-clamp recordings in combination with local activation of synapses at specific locations (we note that injection of a high-osmolarity sucrose solution rather than electrical stimulation has been used in most such experiments, probably because it allows better control of the location and strength of the stimulation). Somatic recordings alone would not be sufficient to measure the true synaptic currents and conductances due to space clamp issues and the (unknown) attenuation of synaptic currents between the synapse and the soma. On the other hand, the necessary dendritic recordings (especially from thin distal dendrites) would be technically extremely challenging, for which only a few laboratories have the necessary expertise available. Therefore, we decided to use approximate values for the synaptic conductances in our study, which led to somatic postsynaptic currents and potentials that are in agreement with experimentally measured responses (see Methods: Modeling: Model testing section of the manuscript). Nevertheless, the models (which we will also share publicly) can be easily updated and re-run if more precise data on these synaptic conductances become available.

2. The here provided single cell models would be ideally suited to examine in more detail differences in the temporal and spatial synaptic integration of excitatory inputs and the influence of inhibition on the dendritic integrative processes in dependence of dendritic location. The impact of inhibition on the very distal dendrites of OLM cells should be stronger than at more proximal sides. Based on Figure 8, the E/I ratio seems to switch between proximal and distal dendrites of OLM cells but appears to be constant in HL neurons. For precise investigations of the role of inhibition in the integration of EPSPs it is of course important to have good estimates on the reversal potential of IPSCs.

We share the Reviewer's interest in all of these functional issues, and hope that, eventually, the data produced in this study will contribute to answering these important questions. The primary goal of the present *Resources* article is to describe our multi-modal dataset that we hope will be utilized in various modeling studies and inspire further experiments. We believe that a comprehensive modeling study of spatio-temporal synaptic integration and action potential generation in the two cell types would deserve at least one full paper. Nevertheless, we already showed in the previous version of the paper how our data can be used to construct morphologically detailed models and to test some of the consequences of the experimentally measured synaptic distributions. We have now added several new modeling results that demonstrate additional uses of our experimental data. Most importantly, we now present fully active models of OLM and HS cells, and characterize their spike output in response to current injections and random as well as spatially organized and temporally patterned synaptic inputs (see Results sections: Modeling spiking responses in OLM and HS neurons: model construction; Modeling the responses of active neurons to synaptic inputs; Supplementary Figures 8, 9, 10, and 11). We have also examined one of the functional consequences of the location-dependence of the E/I balance in our passive models in the simulated high-conductance state, showing that the local equilibrium potential in the dendrites has a distinct dependence on the distance from the soma in the two cell types (Supplementary Figure 7).

3. As stated by the authors in the discussion, more information on the impact of the synaptic input distribution on the recruitment of HL / OLM cells would be perfect. May be the authors see a possibility to make some assumptions on the distribution of voltage gated conductances similar to published data on OLM cells; although the reviewer fully understands the difficulty on making such assumptions and the difficulty to do the related experiments.

Following the suggestion of the Reviewer, we have now constructed and simulated active models of both cell types (Results section: Modeling spiking responses in OLM and HS neurons: model construction). More specifically, we added a variety of voltage-gated conductances to our models based on earlier modeling studies by the group of Frances Skinner, and tuned their maximal conductance values by fitting the responses to a series of depolarizing and hyperpolarizing current steps using features extracted from our physiological recordings. Next, we validated these active models using additional features (Supplementary Figures 8 and 9). Then we examined the action potential output of the models in response to synaptic inputs, varying the number and location of the inputs, and also looked at how the spike output is modulated by time-varying input rates (Results Section: Modeling the responses of active neurons to synaptic inputs; Supplementary Figures 10 and 11).

**Reviewer: 2**

The paper by Takács et al., titled "Synaptic and dendritic architecture of two types of hippocampal somatostatin interneurons", describes extensive profiling of two cell types present among the somatostatin (SST) positive inhibitory neurons in the hippocampus, termed OLM and HS interneurons. The authors characterize the abundance of these two neuron types among the SST interneurons. They quantify the morphologies of the two cell types, including dendritic and axonal features and synaptic targeting, for both the incoming and outgoing synapses. Further, intrinsic electrophysiology of these cells is assessed in slice recordings. Finally, the authors fit morphologically detailed models (but, as they describe them, biophysically simplified, since active conductances are excluded) for these two cell types and investigate responses of the cell models to synaptic inputs.

Overall, the paper has many appealing characteristics. It summarizes a large body of carefully obtained data. The data are multimodal, i.e., come from a variety of experimental techniques to shed light onto multiple properties of the cells. And, modeling is used to integrate the data and obtain further insights through simulations. The paper is well written and clear.

*We are grateful to Reviewer 2 for their constructive comments and hope that the additional computational work included in this revision further demonstrates the potential of this large dataset.*

There are, however, some cons as well. To me, the major issue is that modeling did not use active conductances, therefore resulting in models that cannot generate action potentials and cannot be used for any studies beyond those using weak subthreshold stimuli. For a relatively high-profile publication, this is not sufficient, especially because the paper did not build models for that many cells (only 4 each for OLM and HS cells), and neither did it profile electrophysiology in a particularly large number of cells.
I would therefore suggest that, to make their paper sufficiently strong, the authors should add models with active conductances in the soma (and, perhaps, the axon initial segments). I understand that adding active conductances in dendrites would be way too computationally expensive, so I am not suggesting that. But, developing models with active conductances in the soma would be a necessary addition, which would improve the paper drastically.

Following the suggestion of the Reviewer, we have now constructed and simulated active models of both cell types (Results section: Modeling spiking responses in OLM and HS neurons: model construction). We decided to include voltage-gated conductances in all compartments of the model neurons. More specifically, we added a variety of voltage-gated conductances to our models based on earlier modeling studies of OLM cells by the group of Frances Skinner, and (in the absence of specific data) we assumed that the same set of conductances was present in HS cells as well. We then tuned the maximal conductance values of the voltage-gated channels by fitting the responses of each model neuron to a series of depolarizing and hyperpolarizing current steps using features extracted from our physiological recordings of the two cell types.

With such an addition, the authors can simulate spiking responses to current injections into the soma as well

as to synaptic inputs, for example, at theta frequencies. These simulation results will show whether OLM and HS cells exhibit the same or distinct spiking responses under such circumstances, which will be an important contribution of the modeling. As it stands now, the differences observed with modeling subthreshold phenomena are all rather minor and are likely not highly relevant for physiological situations where cells produce many action potentials.
Obviously, we don't know whether the results will be different between the OLM and HS, but it is important to explore this. That will elevate the results of the paper from less relevant to in vivo physiology to highly relevant.

We first examined the responses of the active models to somatic current injections and compared them with the experimental data using a large number of electrophysiological features, including many that had not been used during the parameter optimization process (Supplementary Figures 8 and 9).
Next, we investigated the action potential output of the models in response to synaptic input, varying the number and location of the inputs (Results section: Modeling the responses of active neurons to synaptic inputs; Supplementary Figure 10).
Finally, we also looked at how the spike output of the models is modulated by scattered synaptic input whose rate varies at the theta frequency (Results section: Modeling the responses of active neurons to synaptic inputs; Supplementary Figure 11).
Interestingly, our simulations showed substantial variability in spiking responses within each cell type, but no major difference was found between the two cell types.

Other comments.

Page 5, bottom, where synapses onto the OLM and HS cells are characterized:
It would be interesting to know how many (what fraction) of the excitatory input synapses are targeting spines vs. shaft and soma, and the same for inhibitory input synapses.

We are grateful for this suggestion, as it has resulted in an interesting set of additional data, which is now presented in the Results section under the title: "Distribution *of glutamatergic and GABAegic synapses on dendritic shafts and spines*". In addition, it has also generated further calculations in the Results section under the title: "*Modeling synaptic distributions in OLM and HS neurons*".

Page 9, Proportions of OLM and HS cells within the somatostatin-positive interneurons of CA1 str. oriens:
It would be good to provide numbers for each mouse, since it's only 3 in each case. One wonders whether the proportions are well conserved across animals.

Now we provided the proportions for each mouse in the text under the subheading *"Proportions of OLM and HS cells within the somatostatin-positive interneurons of CA1 str. oriens"* and we also added these data to the figure legends of Supplementary Fig. 2.

Page 11, modeling subthreshold responses:
The authors only show summary statistics for modeling subthreshold responses to negative current injections in these cells. It would be useful to show in the main figures examples of the actual simulated traces, overlapped with the experimental traces.

The reason why we do not normally show and compare individual voltage traces from experiments and models is that, due to the way we fit our models, such comparisons are not completely straightforward and even potentially misleading. First, we actually use extracted features rather than raw voltage traces to compare model outputs to experimental data during the optimization, so we think that showing this comparison in the figures is a better representation of the quality of the results than plotting the raw traces. Second, the target of the optimization is not a single measurement from one cell, but multiple repeated measurements from a group of cells, and we use the statistics of the extracted features (their means and standard deviations in particular) to define the error function for the optimization. Third, we perform multiple runs of the optimization with different random seeds to obtain a population of model neurons in

each case. We found it more informative to represent this variability in the experimental data and the behavior of the models using the feature statistics.
Nevertheless, we agree with the Reviewer that a visual representation of the traces also carries useful information, so we have added examples of simulated traces (from our new active models) to Figure 7.

Page 13, last paragraph:
The part about mitochondria is interesting, but more information should be shown if the authors want to include it in the paper. There should be a reference to the data on the volume occupied by mitochondria in the Tables (which are mentioned earlier, but readers would forget by now).

Now we added references in the text to the data tables: proportion of mitochondria in Table 1 and Supplementary Table 2, 3.

And, where the authors say that they did not see a change in the behavior of model neurons, they should show these results in a figure or table.

Plotting the results of simulations that included mitochondrial volume produced figures that looked virtually identical on paper to the results of the original simulations, so we decided not to include them in the paper.

Page 14, first paragraph:
These are good references, but the authors completely ignore a large body of work from the Allen Institute, which provided extensive data for constructing detailed models as well as delivered such models. The data are widely used in the community. Some of the relevant papers are as follows:
https://doi.org/10.1038/s41467-017-02718-3
https://doi.org/10.1038/s41593-019-0417-0
https://doi.org/10.1016/j.cell.2020.09.057
https://doi.org/10.1126/science.abj5861
https://doi.org/10.1016/j.neuron.2020.01.040
The same point applies to the statement and references at the very end of Discussion.

Thank you, we agree that these studies offer excellent examples of data-driven models. We have added these references to the text.

Page 19, lines 12-25:
A good starting point can be to use the same ionic conductances for the OLM cells and HS cells. As the recordings presented here show, the electrophysiological responses of these two cell types are not drastically different, suggesting that same conductances might work fine.

We thank the Reviewer for this suggestion, which we followed in the modeling work.

Fig. 1L:
"Complete Neurolucida reconstruction of OLM and HS dendritic trees and (Fig. 5, 6)."
It looks like some text is missing after the "and".

Yes, we have corrected it.

Fig. 9:
Please provide units for Density.

We have corrected it.

Table 4. "Physiological and model parameters and statistics":
These do not seem to be model parameters, or maybe I don't understand something.

The table indeed shows statistics for the physiological features extracted from our experimental recordings. The error has been corrected.

Supplementary Figure 4:
It is somewhat surprising to see such different values of specific capacitance. I would expect them to be closer to 1 uF/cm^2, but that is the case only for one of the 8 cells. For the 4 HS cells, the specific capacitance tends to be small, around 0.6 uF/cm^2.
Are authors sure that these are correct/realistic values? Could they try building their models with the specific capacitance fixed at 1.0 uF/cm^2 and see if they still can reproduce the experimental electrophysiology?
In any case, it would be important to comment on such distinct values of specific capacitance and explain why this is reasonable. It is hard to see what properties of the cell membrane would make the specific capacitance so different (by a factor more than 2 between some of the cells).

This is indeed an issue that deserves further discussion. First, we do not believe that differences in fitted values of the specific membrane capacitance necessarily reflect real variability in this biophysical parameter, especially since most direct measurements resulted in highly conserved values near 1 uF/cm^2. On the other hand, the absolute membrane capacitance of neurons can vary quite a lot, mainly as a result of differences in the extent of the dendritic arbor and a corresponding variability in the membrane surface. Morphological reconstructions based on light microscopy have limited resolution, which could introduce systematic biases in the membrane surface. Therefore, we have found it useful to add the specific capacitance as a tunable parameter to our models, which allows us to compensate for possible biases in the morphological reconstructions and improve the models, mainly in terms of matching the time constants of the real neurons (especially for passive responses).
However, we also repeated the fitting of our passive models with a fixed specific capacitance (at 1 uF/cm^2). The results were generally quite similar, but the quality of the fit was a bit worse, in agreement with the argument above.
Finally, the specific capacitance was fixed at 1 uF/cm^2 in the new active models to match the assumptions of the original models by Lawrence et al (2006).

Supplementary Figs. 5 and 6:
Please label the y-axis. And, it would be more intuitive to let more negative numbers to go down rather than go up.

We have implemented the proposed changes.

Supplementary Figure 6:
It is surprising that the Voltage Base for OLM cells is uniformly fit worse than most other electrophysiological features. Why is that, and can authors improve the fits?

In most cases, the optimized models cannot precisely capture all of the target features simultaneously, and implement trade-offs between fitting different features. This is also likely the case here; we could probably improve the fit to the voltage base (e.g., by assigning this feature a higher weight during the optimization), but this would almost certainly impair the fit for the other features.
However, the error of the voltage base is not particularly large here – it is only about 1.5 mV in absolute terms, and (more importantly) the values for the models are mostly within one standard deviation of the experimental mean. The error appears large mainly because of the fine scale of the x axis in this panel.

**Reviewer 3**

In this paper, the authors do an in depth exploration of OLM and HS somatostatin cell types, finding major differences between the density and distribution of their synaptic inputs and mitochondria. They perform 3D reconstructions using EM for dendritic segments etc. Overall, this is an expansive, detailed and careful (e.g., tissue shrinkage considerations) study that is able to uncover distinct differences between these two somatostatin cell types that can help understand their different functional contributions. However, I have

some comments and suggestions below. Overall, this will be a useful resource for the community.

*We are grateful to Reviewer 3 for their constructive comments and hope that the additional computational work included in this revision further demonstrates the potential of this large dataset.*

GENERAL COMMENTS

1) The authors could situate their work a bit more in the expanding literature of different cell types. For example, consider the recent review of Kessaris and Denaxa (Curr Opin Neurobiol 2023) and references therein. That is, they could describe more about OLM cell types overall I thought (e.g., different types mentioned in Harris et al Plos biol 2018). They clearly describe that they identify OLM cells via Chrna2, but this is a subset as they know, and they do compute relative numbers (proportions on page 9), so important to more broadly and completely situate their work regarding these cell types.

Thank you for bringing this to our attention. We added this part to the introduction:
"the expression of certain genes were detected only in subsets of OLM cells, which may suggest dynamically regulated temporal or permanent differences between OLM neurons [25,35]"

We also emphasized in the results (proportions on page 9) that the Chrna2-positive cells are a subgroup of OLM cells: "Since the expression of the Chrna2 gene is not detectable in every anatomically identified OLM cell [62], and the AAV virus we used for identification in this study does not necessarily infect all Cre-positive cells at the injection site, this number should strictly be considered as an absolute minimum value."

2) Please quantify 'reasonably good'(validation on subthreshold) on page 11 - many other details are quantified and stats applied, so not sure why the authors did not provide any quantification of what they considered reasonably good - eyeballing?

We have replaced the previous wording with a more precise characterization of the match between the output of our models and the corresponding data. In particular, we show that the vast majority of the feature values for the passive models are within two standard deviations of the experimental mean value. (See Results section: Modeling subthreshold responses in OLM and HS neurons: model construction)

3) The authors refer to 'predictive equations' on page 10 regarding synaptic distributions, but I could not find them (prediction coefficients etc.). Given the differences obtained, this would seem to be an important aspect to include fully.

The equation that we used to predict the density of synapses in different dendritic sections is given in the Methods (in section "Modeling: synaptic inputs), while the actual coefficients for all types of cells and synapses are given in Supplementary Table 12. We have also added pointers to these in the Results section (Modeling synaptic distributions in OLM and HS neurons, page 10).

4) As noted by the authors, there are large differences in PSPs when in vivo like states are used relative to in vitro. However, it is interesting that the synaptic differences between OLM and HS cells does not seem to be present anymore. Is this a fair comment? If so, I think that this should be commented on, and whether this might imply less functional differences and/or reduce what their functional differences might be? Also along functional lines, while the authors comment on spiking differences between OLM and HS cells, they do seem to have similar mean frequencies, right?

The observations of the Reviewer are quite accurate, and we have amended the relevant sections of the Results along these lines.

MINOR COMMENTS

- Add 'dendrites' to line 39 on page 8, as it is included on line 23.

It is corrected.

- There are more recent OLM cell models that should be cited - Sekulic et al. Frontiers in Cellular Neuroscience 2020 (line 23, page 19).

Thank you, now we cite this reference.
